# DRAM‐4 and DRAM‐5 are compensatory regulators of autophagy and cell survival in nutrient‐deprived conditions

**DOI:** 10.1111/febs.16365

**Published:** 2022-02-04

**Authors:** Valentin J. A. Barthet, Michaela Mrschtik, Elzbieta Kania, David G. McEwan, Dan Croft, James O'Prey, Jaclyn S. Long, Kevin M. Ryan

**Affiliations:** ^1^ Cancer Research UK Beatson Institute Glasgow UK; ^2^ Institute of Cancer Sciences University of Glasgow UK

**Keywords:** autophagy, cell death, DRAM family, nutrient deprivation

## Abstract

Macroautophagy is a membrane‐trafficking process that delivers cytoplasmic material to lysosomes for degradation. The process preserves cellular integrity by removing damaged cellular constituents and can promote cell survival by providing substrates for energy production during hiatuses of nutrient availability. The process is also highly responsive to other forms of cellular stress. For example, DNA damage can induce autophagy and this involves up‐regulation of the Damage‐Regulated Autophagy Modulator‐1 (DRAM‐1) by the tumor suppressor p53. DRAM‐1 belongs to an evolutionarily conserved protein family, which has five members in humans and we describe here the initial characterization of two members of this family, which we term DRAM‐4 and DRAM‐5 for DRAM‐Related/Associated Member 4/5. We show that the genes encoding these proteins are not regulated by p53, but instead are induced by nutrient deprivation. Similar to other DRAM family proteins, however, DRAM‐4 principally localizes to endosomes and DRAM‐5 to the plasma membrane and both modulate autophagy flux when over‐expressed. Deletion of *DRAM‐4* using CRISPR/Cas‐9 also increased autophagy flux, but we found that *DRAM‐4* and *DRAM‐5* undergo compensatory regulation, such that deletion of *DRAM‐4* does not affect autophagy flux in the absence of DRAM‐5. Similarly, deletion of *DRAM‐4* also promotes cell survival following growth of cells in the absence of amino acids, serum, or glucose, but this effect is also impacted by the absence of DRAM‐5. In summary, DRAM‐4 and DRAM‐5 are nutrient‐responsive members of the DRAM family that exhibit interconnected roles in the regulation of autophagy and cell survival under nutrient‐deprived conditions.

## Graphical abstract

DRAM‐4 and DRAM‐5 are two proteins that are members of the DRAM family. Here, we explore the role of DRAM‐4 and DRAM‐5 in autophagy and cell survival. We report that DRAM‐4 prevents autophagy, while DRAM‐5 enhances autophagy. Upon nutrient deprivation, DRAM‐4 loss promotes cell survival via DRAM‐5 upregulation.

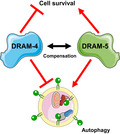

**Abbreviations**
4EBP1: eukaryotic initiation factor 4E‐binding protein
ATG: autophagy‐related genes
DMEM: Dulbecco's Modified Eagle Medium
DRAM‐1: Damage‐Regulated Autophagy Modulator 1
DRAM‐4/5: DRAM‐Related/Associated Member 4/5
EBSS: Earle's Balanced Salt Solution
EEA1: endosome‐associated protein 1
ERK2: extracellular signal‐regulated kinase 2
IFNγ: interferon gamma
LC3: microtubule‐associated proteins 1A/1B light chain 3
mTORC1: mechanistic target of rapamycin complex 1
NF‐κB: nuclear factor kappa‐light‐chain‐enhancer of activated B cells
NSTSMI: non‐ST segment myocardial infarction
TNF: tumor necrosis factor
ULK1: Unc‐51 Like Autophagy Activating Kinase 1

## Introduction

The ability to respond to various forms of cellular stress is an essential facet for cell survival. Cellular stress can come in various forms such as DNA damage, protein damage and periods of nutrient deprivation. Cells and organisms have evolved various mechanisms to deal with the impact of stress including DNA repair and pathways that mediate the degradation of damaged proteins and organelles in either proteasomes or lysosomes.

Autophagy represents a group of processes that serve to deliver cellular material to lysosomes for degradation [1]. There are three major forms of autophagy described to date: macroautophagy, microautophagy, and chaperone‐mediated autophagy. Macroautophagy is the most widely studied and is often (and hereafter) more simply referred to as autophagy. The process is highly phylogenetically conserved and is orchestrated by a core group of autophagy‐related genes (*ATG*) that are conserved from yeast to humans [2]. The process of autophagy is initiated when lipids are sourced from within the cell and engineered by ATG proteins into a double‐membraned structure referred to as the isolation membrane or phagophore [3, 4]. This structure then matures into a double‐membraned ball‐like organelle called an autophagosome that encapsulates cargo destined for degradation [1]. The autophagosome membrane contains a key membrane‐anchored protein termed Microtubule‐associated proteins 1A/1B light chain 3 (LC3). This protein is integral to the process of autophagy as it acts as a tether for adaptor proteins bound to cellular cargo [5]. In cells with low levels of autophagy, LC3 is constitutively cleaved to generate a free, cytoplasmic form, termed LC3‐I. Upon initiation of autophagy, LC3‐I is conjugated to phosphatidylethanolamine to form LC3‐II, which serves as the anchor in the autophagosome membrane [6]. Following closure, autophagosomes can fuse with a variety of cellular organelles including endosomes and multivesicular bodies, but ultimately, fusion occurs with lysosomes to form new organelles termed autolysosomes [1]. Acidic hydrolases provided by the lysosome cause degradation of the contents of the autophagosome in the autolysosome. The constituent parts of the degraded cargo can then be further catabolized to generate energy for survival or recycled into biosynthetic pathways to either generate more of the same cellular components or different components in tissue remodeling situations [1].

While mitigating various forms of stress to maintain cellular homeostasis, it is considered that the primordial function of autophagy is to degrade nonessential cellular constituents to maintain cell survival during periods of nutrient deprivation. This is clearly evident from studies in yeast [7], but also essential in mammals as they bridge the feeding hiatus associated with the switch from feeding in utero to suckling after birth [8].

Autophagy is, however, also highly adaptable and can respond to a variety of intracellular and extracellular events in order to orchestrate specific changes in the cell. In these contexts, additional cellular proteins either regulate or connect with the core ATG machinery to bring about bespoke desired effects. One such protein is Damage‐Regulated Autophagy Modulator 1 (DRAM‐1), which is a lysosomal protein that has been shown to regulate autophagy and the activation of the nutrient‐sensing kinase complex mechanistic target of rapamycin complex 1 (mTORC1) [9, 10]. Studies by ourselves and others have shown that DRAM‐1 is transcriptionally regulated by the tumor suppressor p53 [9] and NF‐kB [11].

In our previous studies, we reported that DRAM‐1 belongs to a protein family that has 5 members in humans [12]. To date, DRAM‐2 (encoded by *TMEM77*) and DRAM‐3 (encoded by *TMEM150B*) have been characterized as being members of this family [12]. DRAM‐2 has also been implicated in retinal dystrophy and non‐ST segment myocardial infarction (NSTSMI) [13, 14]. However, the role of the protein in autophagy is currently unclear, with some studies indicating that DRAM‐2 regulates autophagy in certain contexts [15], but not in others [12]. This indicates potential context‐specific effects of the protein in autophagy and that the impact of DRAM‐2 in retinal dystrophy and NSTMI may or may not be autophagy‐dependent. In contrast to DRAM‐1 and DRAM‐2, which exhibit very high similarity at the peptide level, DRAM‐3 is more diverse. It does, however, modulate autophagy and promotes cell survival when glucose is limited [16]. In this study, we provide an initial characterization of the two other members of the DRAM family in the context of autophagy regulation and what is already known about DRAM‐1, DRAM‐2, and DRAM‐3.

## Results

### Two DRAM1‐related proteins are induced by nutrient deprivation, but not by p53

In our previous work, we reported a family of human proteins related to the autophagy regulator DRAM‐1 [12]. In addition to DRAM‐1, we have also previously characterized two other members of the family, DRAM‐2 and DRAM‐3 [12, 16]. In this study, we now characterize the final two members of the family with regard to our previous findings with DRAM‐1, ‐2, and ‐3. These remaining two family members are encoded by *TMEM150C* and *TMEM150A* and due to their relationship based on amino acid similarity to other DRAM proteins, we respectively refer to them as DRAM‐4 and DRAM‐5 (for DRAM‐Related/Associated Member 4 & 5). It should, however, also be noted that although not previously characterized as DRAM family proteins, DRAM‐4 and DRAM‐5 have previously been respectively described as Tentonin‐3 [17] and the mammalian homologue of yeast Sfk1 [18].

**Fig. 1:**
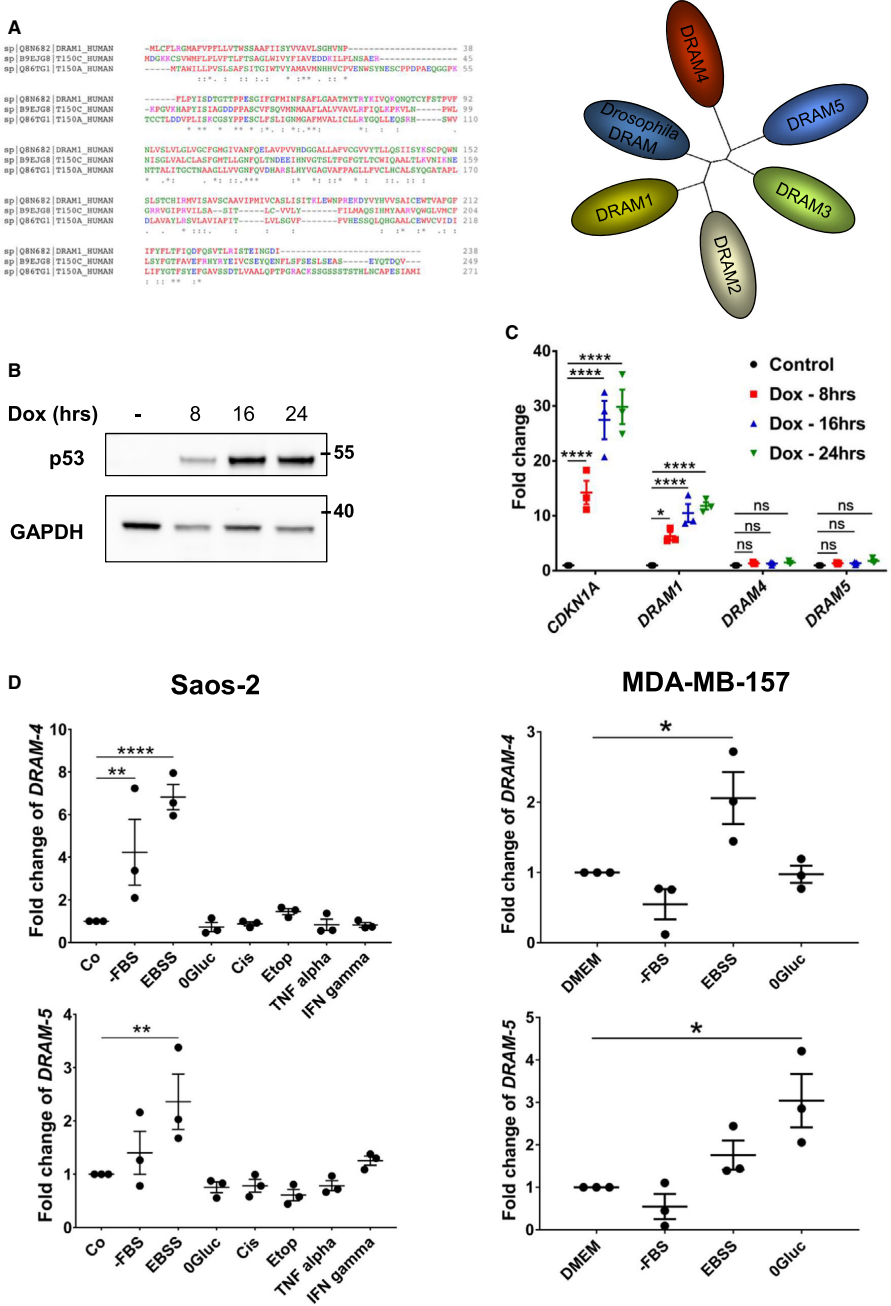
DRAM‐4 and DRAM‐5 are induced by starvation, but not by p53. (A) Sequence alignment between the peptide sequences of DRAM‐1, TMEM150C (DRAM‐4), and TMEM150A (DRAM‐5) (left). Phylogenic tree within the DRAM family (right). (B) Immunoblotting analysis of p53 expression following doxycycline addition for 8, 16, and 24 h in Saos‐2‐TetOn‐p53 cells. GAPDH expression was used as the loading control. (C) Quantitative RT‐PCR analysis of *CDKN1A*, *DRAM1*, *DRAM4 (TMEM150C)*, and *DRAM5 (TMEM150A)* mRNA isolated from Saos‐2‐TetOn‐p53 cells treated with doxycycline for 8, 16, and 24 h. 18S was used as the internal amplification control. Data are means ± SEM of three experiments and were analyzed by one‐way ANOVA with Dunnett correction for multiple comparison tests (*P < 0.05 and ****P < 0.0001). All data points are the mean from technical triplicates. (D) Quantitative RT‐PCR analysis of *DRAM‐4* and *DRAM‐5* mRNA isolated from Saos2 (left) or MDA‐MB‐157 (right) cultured for 24 h in nutrient‐deprived conditions (‐FBS, EBSS, and 0Gluc); or treated for 24 h with DNA damaging agents (Cis: cisplatin; Etop: etoposide) or inflammatory agents (TNFα and IFN‐γ). 18S was used as the internal amplification control. Data are means ± SEM of three independent experiments and were analyzed by one‐way ANOVA with Dunnett correction for multiple comparison tests (*P < 0.05, **P < 0.01, and ****P < 0.0001). All data points are the mean from three technical replicates.

Sequence alignment shows that DRAM‐4 and DRAM‐5 are more similar to each other than they are to other DRAM family members, with 48% similarity at the protein level between DRAM‐4 and DRAM‐5, whereas there is only 38% and 35% similarity to DRAM‐1, respectively (Fig. 1A), indicating as we previously reported that DRAM‐4 and DRAM‐5 form a distinct arm of the DRAM phylogenetic tree (Fig. 1A).

One distinct feature of DRAM‐1 is that it is induced by the tumor suppressor protein p53 [9]. In contrast, neither DRAM‐2 nor DRAM‐3 are regulated in this way [12, 16]. To test whether DRAM‐4 and DRAM‐5 are induced by p53, we utilized a previously described Saos‐2 cell line which contains no endogenous p53, but which expresses a doxycycline‐inducible *TP53* transgene [19]. Treatment of these cells with doxycycline causes the induction of p53 that peaks at 24 h (Fig. 1B). Analysis by qPCR revealed that while this treatment resulted in a significant concomitant increase in *DRAM‐1* and the classic p53 target *p21/CDKN1A*, it did not result in an increase in *DRAM‐4* or *DRAM‐5* mRNA levels (Fig. 1C), indicating that they are not p53 target genes.

As autophagy is induced by nutrient deprivation and because DRAM‐3 protects against glucose deprivation [16], we next tested if *DRAM‐4* and *DRAM‐5* are induced upon depletion of various nutrients. This revealed that in osteosarcoma (Saos‐2) and breast cancer (MDA‐MB‐157) cells, *DRAM‐4* and *DRAM‐5* are induced to varying extents upon culture in media Dulbecco's Modified Eagle Medium (DMEM) lacking glucose (0Gluc), serum (‐FBS), or amino acids and serum (EBSS) (Fig. 1D). Interestingly, in line with the lack of induction by p53, we did neither observe induction of DRAM‐4 or DRAM‐5 following treatment with DNA‐damaging agents (cisplatin or etoposide), nor did we see induction in response to inflammatory stimuli (TNF or IFNγ) as it has previously been shown for DRAM‐1 [11] (Fig. 1D).

### DRAM‐4 and DRAM‐5 localize respectively to endosomes and the plasma membrane

**Fig. 2:**
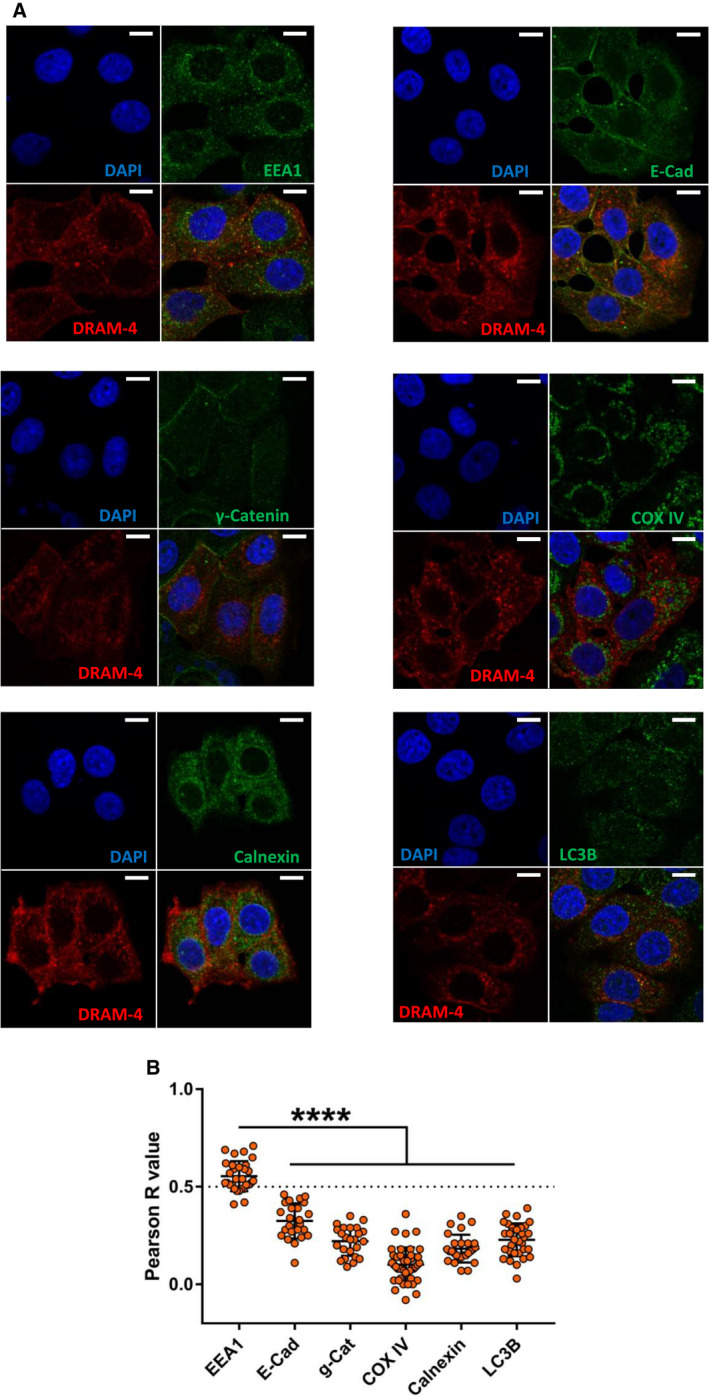
DRAM‐4 localizes with early endosomes. (A) Immunofluorescence analysis of DRAM‐4 expression in Saos‐2 cells overexpressing Myc‐tagged DRAM‐4. Colocalization with the early endosome marker EEA1; the plasma membrane markers E‐Cadherin (E‐Cad) and γ‐catenin (g‐Cat); the mitochondria marker COX IV; the endoplasmic reticulum marker calnexin; and the autophagosome marker LC3B. 4′,6‐diamidino‐2‐phenylindole (DAPI) stains nuclei. Scale bar represents 10 µm. (B) Quantification of colocalization between DRAM‐4 and the different cell compartments from A. Data represents the Pearson R value for colocalization within one cell. Data are means ± SD of three independent experiments (*n* = 24 for E‐Cad and g‐Cat, 30 for EEA1, 44 for COX IV, 27 for Calnexin, and 32 for LC3B). The dash line at *R* = 0.5 indicates the accepted threshold for colocalization (*R* ≥ 0.5 for colocalization). Data were analyzed by one‐way ANOVA with Dunnett correction for multiple comparison tests (****P < 0.0001).

**Fig. 3:**
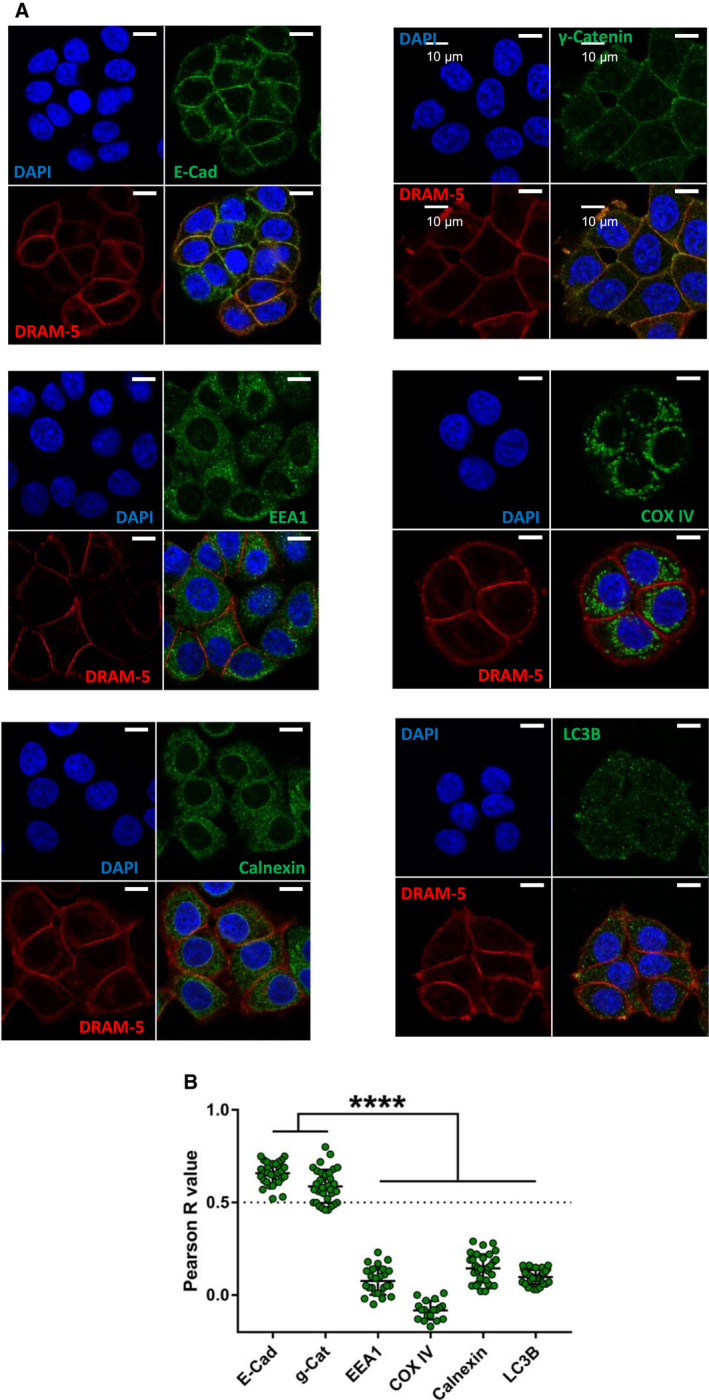
DRAM‐5 localizes with adherens and cell–cell junctions. (A) Immunofluorescence analysis of DRAM‐5 expression in Saos‐2 cells overexpressing Myc‐tagged DRAM‐5. Colocalization with the early endosome marker EEA1; the plasma membrane markers E‐Cadherin (E‐Cad) and γ‐catenin; the mitochondria marker COX IV; the endoplasmic reticulum marker calnexin; and the autophagosome marker LC3B. 4′,6‐diamidino‐2‐phenylindole (DAPI) stains nuclei. Scale bar represents 10 µm. (B) Quantification of colocalization between DRAM‐5 and the different cell compartments from A. Data represents the Pearson *R* value for colocalization within one cell. Data are means ± SD of three independent experiments (*n* = 31 for E‐Cad, 33 for g‐Cat, 25 for EEA1, 18 for COX IV, 33 for Calnexin, and 34 for LC3B). The dash line at *R* = 0.5 indicates the accepted threshold for colocalization (*R* ≥ 0.5 for colocalization). Data were analyzed by one‐way ANOVA with Dunnett correction for multiple comparison tests (****P < 0.0001).

We previously found that DRAM‐1 predominantly localizes to lysosomes [9], DRAM‐2 to lysosomes and endosomes [12], and DRAM‐3 to lysosomes, endosomes and actin‐rich focal adhesions at the plasma membrane [16]. These data indicate that if DRAM family members have similar functions then they potentially execute these functions at different locations within the cell. We were therefore naturally interested to know the cellular localization of DRAM‐4 and DRAM‐5. To examine this, we co‐stained cells expressing either Myc‐tagged DRAM‐4 or DRAM‐5 with markers for autophagosomes (LC3), endosomes (EEA1), mitochondria (COX IV), the endoplasmic reticulum (calnexin), and the plasma membrane (E‐Cadherin and γ‐catenin). This revealed that DRAM‐4 primarily colocalized with EEA1 (Fig. 2A,B) and DRAM‐5 colocalized with E‐Cadherin and γ‐Catenin (markers of the plasma membrane) (Fig. 3A,B). No colocalization however was found at other cellular locations (Figs 2, 3A,B and 2, 3A,B).

### DRAM‐4 and DRAM‐5 are highly expressed in breast cancer cells and regulate autophagy

**Fig. 4:**
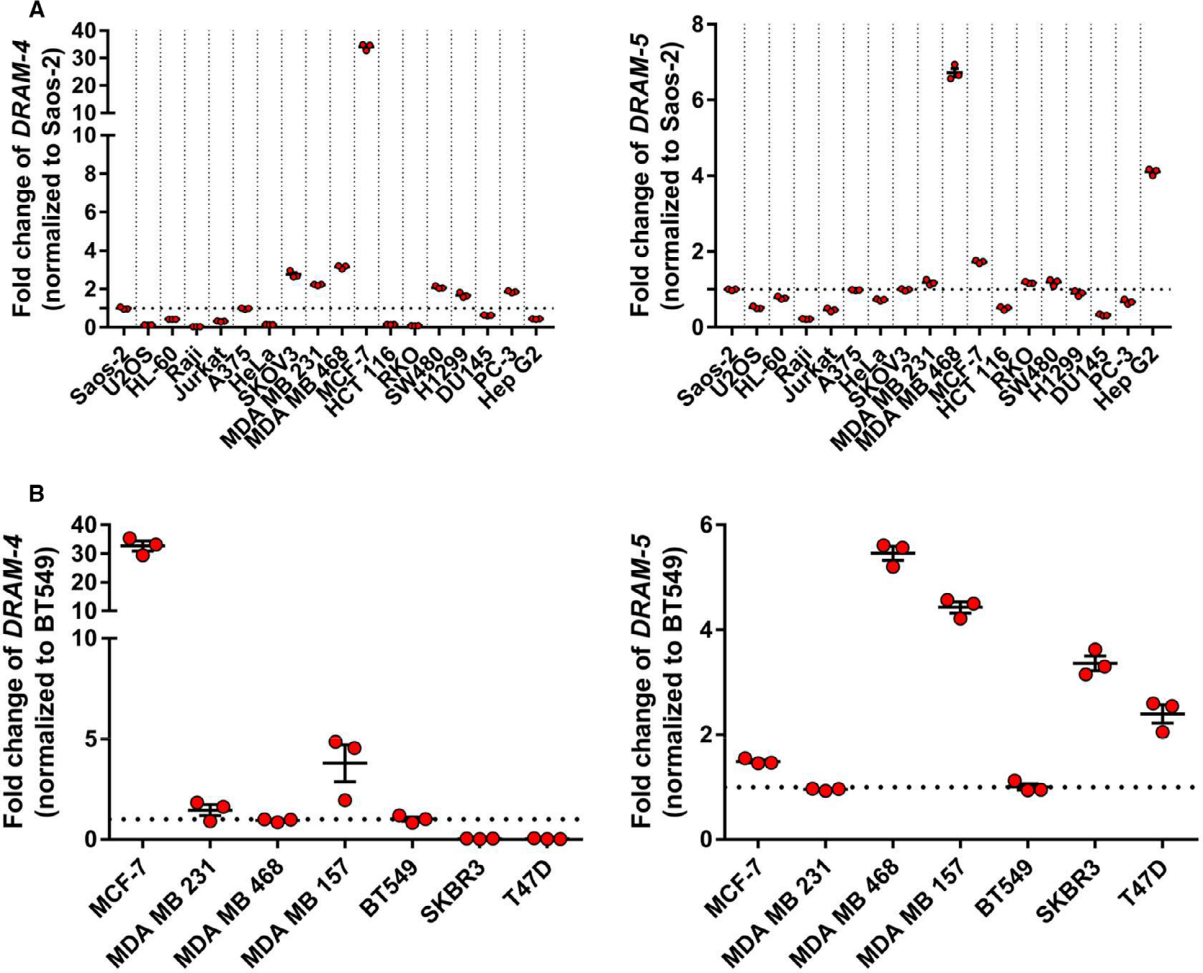
*DRAM‐4* and *DRAM‐5* are highly expressed in breast cancer cells. (A) Quantitative RT‐PCR analysis of *DRAM‐4* (left) and *DRAM‐5* (right) mRNA isolated from different human cancer cell lines. Data are means ± SEM of three independent experiments. All data points are the mean from technical triplicates. 18S was used as the internal amplification control. The fold change was normalized relative to Saos‐2 expression. (B) Quantitative RT‐PCR analysis of *DRAM‐4* (left) and *DRAM‐5* (right) mRNA isolated from different human breast cancer cell lines. Data are means ± SEM of three independent experiments. All data points are the mean from technical triplicates. 18S was used as the internal amplification control. The fold change was normalized relative to BT549 expression.

In order to understand the functional roles of DRAM‐4 and DRAM‐5, we wanted to know in which tissue or cell types they are predominantly expressed. We therefore examined mRNA levels for *DRAM‐4* and *DRAM‐5* in a panel of cell lines from a variety of tissues/tumor types. This showed that while the expression of both genes was relatively low in most cells, the expression of *DRAM‐4* was higher in MCF‐7 and *DRAM‐5* was higher in MDA‐MB‐468, with moderate level of expression in HepG2 cells (Fig. 4A). As MCF‐7 and MDA‐MB‐468 are both derived from breast cancers, we next examined the expression of *DRAM‐4* and *DRAM‐5* in a broader panel of breast cancer cell lines, which revealed that *DRAM‐4* and *DRAM‐5* are expressed to varying levels across the panel (Fig. 4B).

**Fig. 5:**
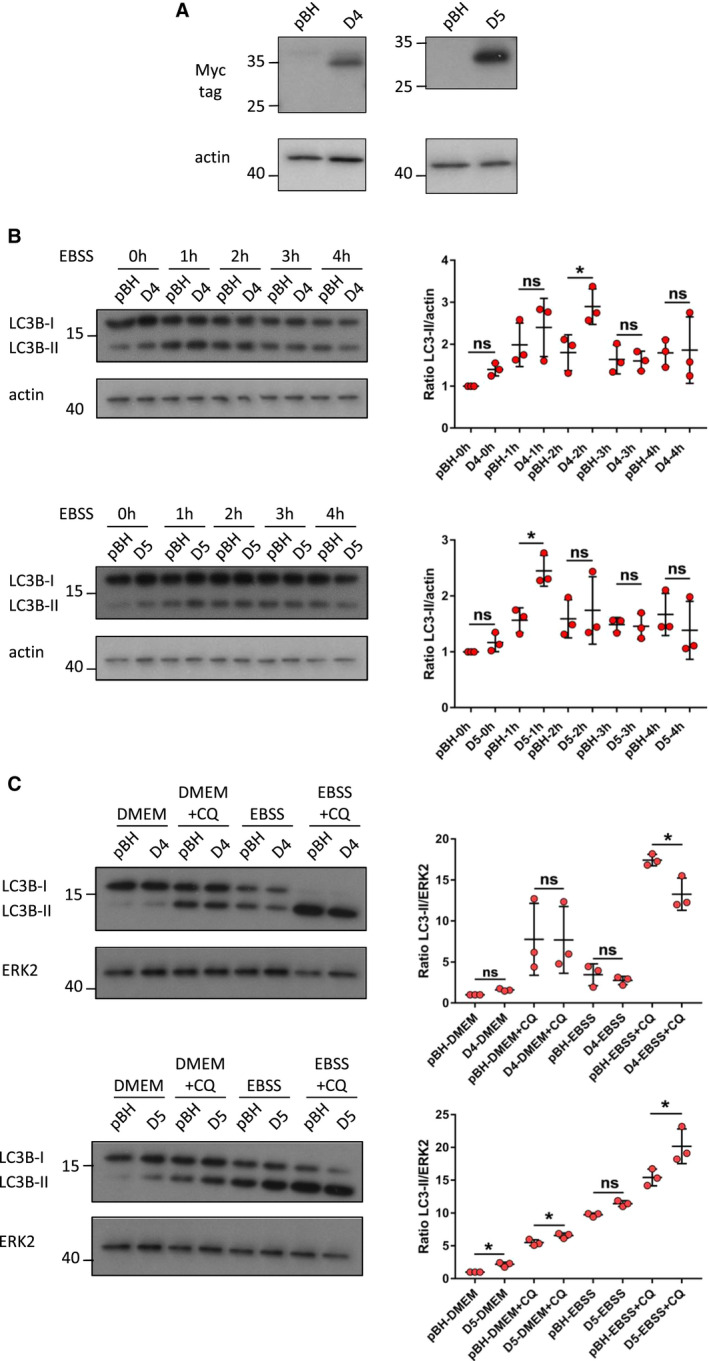
DRAM‐5, but not DRAM‐4, enhances basal and starvation‐induced autophagic flux. (A) Immunoblotting analysis of Myc‐tagged DRAM‐4 (left) and Myc‐tagged DRAM‐5 (right) expression in Saos‐2 cells overexpressing Myc‐tagged DRAM‐4 (D4), Myc‐tagged DRAM‐5 (D5), or the empty control (pBH). Actin expression was used as the loading control for each blot. (B) (Left) Immunoblotting analysis of LC3B‐I and LC3B‐II expression in Saos‐2 cells overexpressing either Myc‐tagged DRAM‐4 (D4) (top) or Myc‐tagged DRAM‐5 (D5) (bottom) starved for either 0, 1, 2, 3, or 4 h in EBSS. Actin expression was used as the loading control for each blot. (Right) Quantification of the corresponding immunoblots. Data are means ± SD of three independent experiments and were analyzed by one‐way ANOVA with Sidak correction for multiple comparison tests (*P < 0.05). (C) (Left) Immunoblotting analysis of LC3B‐I and LC3B‐II expression in Saos‐2 cells overexpressing either Myc‐tagged DRAM‐4 (D4) (top) or Myc‐tagged DRAM‐5 (D5) (bottom) cultured for 2 h (DRAM‐4) or 1 h (DRAM‐5) in the presence/absence of 5 μm of chloroquine (CQ) under normal conditions (DMEM) or starved conditions (EBSS). ERK2 expression was used as the loading control. (Right) Quantification of the corresponding immunoblots. Data are means ± SD of three independent experiments and were analyzed by one‐way ANOVA with Sidak correction for multiple comparison tests (*P < 0.05).

We chose to focus our further studies on one cell line that had comparatively average levels of mRNA for *DRAM‐4* and *DRAM‐5* – Saos‐2, and one cell line that had higher levels of both *DRAM‐4* and *DRAM‐5* – MDA‐MB‐157. We then used these cells to examine the effects of DRAM‐4 and DRAM‐5 over‐expression on autophagy and cell survival. In the first instance, we over‐expressed DRAM‐4 and DRAM‐5 via retroviral transduction of Saos‐2 cells (Fig. 5A). These cells were then incubated for up to 4 h in either replete culture medium (DMEM) or Earle’s balanced salt solution (EBSS), which lacks amino acids and is a well characterized inducer of autophagy. In cells over‐expressing either DRAM‐4 or DRAM‐5, higher levels of LC3‐II (the form of LC3 associated with autophagosomes) were observed in cells cultured in replete medium, when compared to cells expressing empty retroviral vector as control (Fig. 5B). This difference was significantly different after culture for 1 h in EBSS for DRAM‐5 and after culture for 2 h in EBSS for DRAM‐4, but the difference was lost at later time points when all cell lines exhibited higher levels of LC3‐II, as would be expected upon culture in EBSS (Fig. 5B).

As autophagosomes and, by association, the conjugation of LC3 with phosphatidylethanolamine to form LC3‐II, are transient midpoints in autophagy, the accumulation of LC3‐II can mean either that autophagy is being induced or that autophagy has been impeded at a stage distal to LC3‐II formation, leading to its accumulation. To discern between these two possibilities with regard to the accumulation of LC3‐II following over‐expression of DRAM‐4 or DRAM‐5, we incubated cells in the lysosomotropic agent chloroquine, which blocks the turnover stage of autophagy. In the presence of chloroquine, events that cause induction of autophagy will increase LC3‐II accumulation greater than that seen with chloroquine alone. In contrast, events that impede the turnover stage of autophagy to cause an increase in LC3‐II, will have no effect over and above the accumulation caused by blocking turnover with chloroquine. Using this approach, we found that cells overexpressing DRAM‐5, but not DRAM‐4, exhibited a greater accumulation of LC3‐II in the presence of chloroquine when compared to cells expressing the vector control (Fig. 5C). This suggests that DRAM‐5 over‐expression promotes autophagy and that DRAM‐4 overexpression impedes autophagy at a point subsequent to LC3 conjugation.

**Fig. 6:**
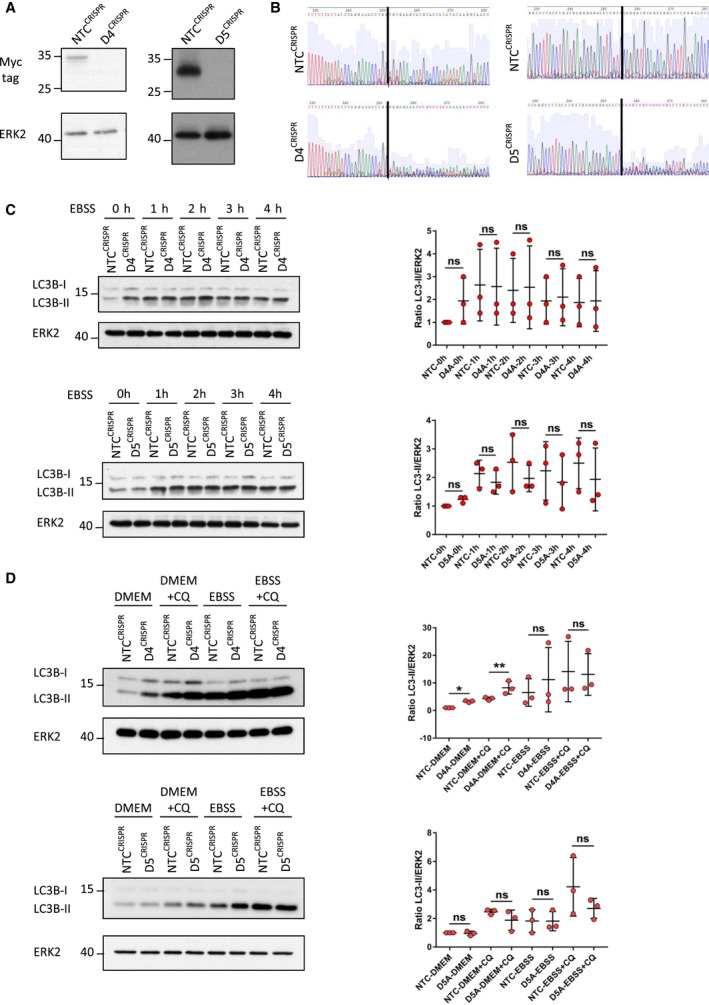
DRAM‐4 deletion, but not DRAM‐5 deletion, enhances basal autophagic flux in breast cancer cells. (A) Immunoblotting analysis of Myc‐tagged DRAM‐4 (left) and Myc‐tagged DRAM‐5 (right) expression in lentiCRISPR MDA‐MB‐157 cells for either nontargeting control (*NTC^CRISPR^*), *DRAM‐4* (*D4^CRISPR^*), or *DRAM‐5* (*D5^CRISPR^*) that have been transfected with DRAM‐4‐Myc or DRAM‐5‐Myc, accordingly. ERK2 expression was used as the loading control. (B) Genomic DNA from Saos‐2 cells infected with lentivirus containing control (NTC) or DRAM‐4‐ or DRAM‐5‐targeting constructs was sequenced for CRISPR‐induced DNA frameshifts. (C) (Left) Immunoblotting analysis of LC3B‐I and LC3B‐II expression in *D4^CRISPR^* (top) and *D5^CRISPR^* (bottom) MDA‐MB‐157 cells starved for either 0, 1, 2, 3, or 4 h in EBSS. ERK2 expression was used as the loading control. (Right) Quantification of the corresponding immunoblots. Data are means ± SD of three independent experiments and were analyzed by one‐way ANOVA with Sidak correction for multiple comparison tests. (D) (Left) Immunoblotting analysis of LC3B‐I and LC3B‐II expression in *D4^CRISPR^* (top) and *D5^CRISPR^* (bottom) MDA‐MB‐157 cells cultured for 2 h in the presence/absence of 5 μm of chloroquine (CQ) under normal conditions (DMEM) or starved conditions (EBSS). ERK2 expression was used as the loading control. (Right) Quantification of the corresponding immunoblots. Data are means ± SD of three independent experiments and were analyzed by one‐way ANOVA with Sidak correction for multiple comparison tests (*P < 0.05; **P < 0.01).

We were interested to know if the effects we observed by over‐expressing DRAM‐4 and DRAM‐5 could also be seen when the expression of the endogenous genes was reduced. To do this, we utilized MDA‐MB‐157 cells expressing relatively high levels of DRAM‐4 and DRAM‐5 compared to other cell lines, and disrupted *DRAM‐4* and *DRAM‐5* expression in these cells using CRISPR/Cas‐9 (Fig. 6A,B). This revealed that deletion of *DRAM‐5* had no impact on the levels of LC3‐II either under basal conditions or following incubation in EBSS (Fig. 6C,D). In contrast, cells in which *DRAM‐4* was deleted often had higher levels of LC3‐II at basal levels when compared to cells infected with nontargeting CRISPR control (Fig. 6C,D). Moreover, by using chloroquine to inhibit the turnover stage of autophagy, we found that these increased levels were significantly and reproducibly observed, indicating that the loss of DRAM‐4 causes an induction of autophagy under basal, but not prolonged starvation conditions (Fig. 6D).

### DRAM‐4 and DRAM‐5 exhibit compensatory effects on autophagy and cell survival

**Fig. 7:**
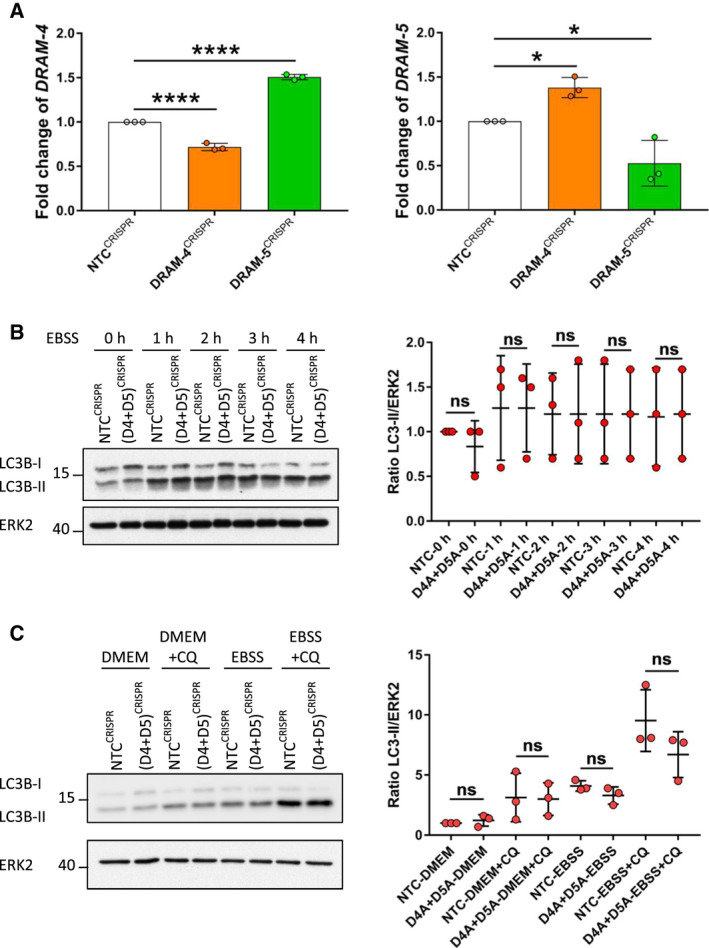
Loss‐of‐DRAM‐4‐induced autophagic flux is triggered by a compensation mechanism involving DRAM‐5. (A) Quantitative RT‐PCR analysis of *DRAM‐4* (left) and *DRAM‐5* (right) mRNA isolated from *NTC^CRISPR^*, *D4^CRISPR^*, and *D5^CRISPR^* MDA‐MB‐157 cells. Data are means ± SEM of three independent experiments and were analyzed by one‐way ANOVA with Dunnett correction for multiple comparison tests (*P < 0.05 and ****P < 0.0001). All data points are the mean from three technical replicates. 18S was used as the internal amplification control. The fold change was normalized relative to *NTC^CRISPR^* expression. (B) (Left) Immunoblotting analysis of LC3B‐I and LC3B‐II expression in *NTC^CRISPR^* and (*D4+D5*)*^CRISPR^* MDA‐MB‐157 cells starved for either 0, 1, 2, 3, or 4 h in EBSS. ERK2 expression was used as the loading control. (Right) Quantification of the corresponding immunoblots. Data are means ± SD of three independent experiments and were analyzed by one‐way ANOVA with Sidak correction for multiple comparison tests. (C) (Left) Immunoblotting analysis of LC3B‐I and LC3B‐II expression in *NTC^CRISPR^* and (*D4+D5*)*^CRISPR^* MDA‐MB‐157 cells cultured for 2 h in the presence/absence of 5 μm of chloroquine (CQ) under normal conditions (DMEM) or starved conditions (EBSS). ERK2 expression was used as the loading control. (Right) Quantification of the corresponding immunoblots. Data are means ± SD of three independent experiments and were analyzed by one‐way ANOVA with Sidak correction for multiple comparison tests.

In our studies with over‐expression of DRAM‐4 and DRAM‐5, we found that over‐expression of either protein caused an increase in the levels of LC3‐II (Fig. 5B). Subsequently, we showed DRAM‐5 expression promoted autophagy, whereas over‐expression of DRAM‐4 expression caused a block to autophagy at a stage post formation of autophagosomes (Fig. 5C). As a result, it was surprising to find that deletion of *DRAM‐4* using CRISPR/Cas‐9 caused an increase in the initiation of autophagy. As DRAM‐4 and DRAM‐5 are highly related at the peptide levels (Fig. 1A), this caused us to consider whether DRAM‐5, which we had shown promotes autophagy (Fig. 5C), may undergo a compensatory gene up‐regulation upon loss of *DRAM‐4*. To test this hypothesis, we measured the levels of *DRAM‐4* and *DRAM‐5* mRNAs in cells where either *DRAM‐4* or *DRAM‐5* has been deleted by CRISPR/Cas‐9, which showed reciprocally that *DRAM‐5* is up‐regulated upon loss of *DRAM‐4* and vice versa (Figs 6A,B and 7A).

These findings caused us to question if the up‐regulation of autophagy seen upon deletion of *DRAM‐4* was due to an increase in DRAM‐5, which we had shown induces autophagy when its expression is increased ectopically (Fig. 5B,C). We therefore generated DRAM‐4 and DRAM‐5 double knock out MDA‐MB‐157 cells and examined the levels of LC3‐II following culture in EBSS in either the absence or presence of chloroquine. In agreement with this idea, we observed that the cells deleted for both *DRAM‐4* and *DRAM‐5* did not show increased levels of LC3‐II at either basal conditions or following culture in EBSS when compared with the cells expressing nontargeting CRISPR controls (Fig. 7B). Moreover, no change in autophagic flux was observed in the cells exposed to chloroquine in which *DRAM‐4* and *DRAM‐5* had been deleted when compared to cells expressing nontargeting CRISPR controls (Fig. 7C). These results are in contrast to what we observed upon deletion of *DRAM‐4* alone and indicate that deletion of *DRAM‐4* promotes autophagy due to up‐regulation of DRAM‐5.

**Fig. 8:**
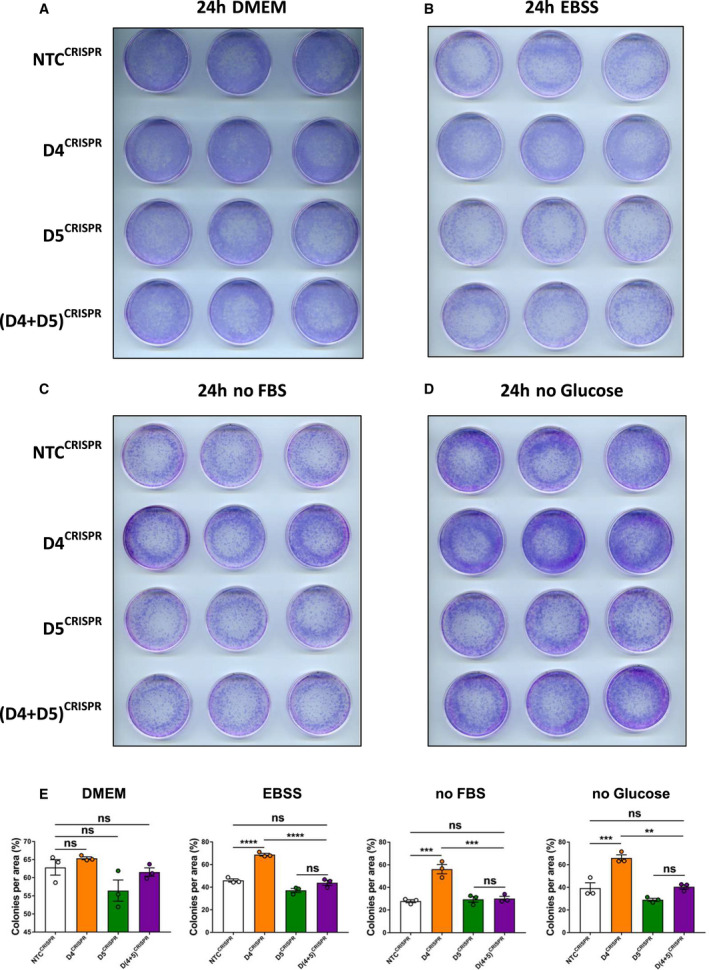
Loss of DRAM‐4 leads to enhanced survival upon starvation in a DRAM‐5‐dependent manner. (A–D) Clonogenic assays of *NTC^CRISPR^*, *D4^CRISPR^*, *D5^CRISPR^*, and (*D4+D5*)*^CRISPR^* MDA‐MB‐157 cells cultured for 24 h in either DMEM (A), EBSS (B), serum‐free media (no FBS – C), and glucose‐free media (D) and then cultured back in DMEM for 7–10 days. Each row represents the technical triplicates for the representative biological replicate. (E) Quantification from A–D. Data are means ± SEM of three independent experiments and were analyzed by one‐way ANOVA with Dunnett correction for multiple comparison tests (**P < 0.01, ***P < 0.001, and ****P < 0.0001). All data points are the mean from three technical replicates.

Being as we found that DRAM‐4 and DRAM‐5 are both up‐regulated in response to nutrient depletion (Fig. 1D) and because autophagy promotes survival under nutrient‐deprived conditions, we reasoned that DRAM‐4 and DRAM‐5 may also have roles in regulating cell survival when nutrients are limited/restricted. To explore this possibility, MDA‐MB‐157 cells lacking either DRAM‐4, DRAM‐5, or DRAM‐4 and DRAM‐5 were cultured in replete DMEM, EBSS, or DMEM lacking either serum or glucose. After 24 h, cells were re‐seeded back into replete medium and left to grow for 7–10 days before the impact on the clonogenic potential of the cells was assessed by Giemsa staining. For the cells in replete DMEM, this showed no effect of *DRAM‐4* deletion (Fig. 8A,E). In contrast, deletion of *DRAM‐4* promoted the clonogenic potential of the cells cultured in either EBSS, the absence of serum or the absence of glucose (Fig. 8B–D,E).

As we had found that the induction of autophagy in response to *DRAM‐4* deletion could be reversed by co‐deletion of *DRAM‐5* (Fig. 7B,C), we finally questioned whether the pro‐survival effects of *DRAM‐4* deletion in EBSS, the absence of serum or the absence of glucose might also be mediated by a compensatory up‐regulation of *DRAM‐5*. We considered this possible, as neither DRAM‐4 nor DRAM‐5 is up‐regulated in response to glucose deprivation in the cells where neither gene was disrupted by CRISPR/Cas9. To test this, we compared the clonogenic potential of *DRAM‐4*‐deleted cells to cells in which *DRAM‐4* and *DRAM‐5* had been co‐deleted following culture in either EBSS, and medium lacking serum or glucose. Similar to our findings regarding autophagy, these studies also showed that the increased clonogenic capacity conferred by deletion of *DRAM‐4* was lost upon the deletion of *DRAM‐5* (Fig. 8B–E), underscoring the intricate connection between these two closely related proteins.

In summary, we report here the initial characterization of two new members of the DRAM family of proteins. Like other DRAM proteins, we found that DRAM‐4 and DRAM‐5 can regulate autophagy and have roles in promoting cell survival in nutrient‐depleted states. Moreover, the compensatory regulation and interdependency between both proteins in the promotion of autophagy and cell survival suggest similar overlapping roles even though the proteins are present at different locations within the cell.

## Discussion

**Fig. 9:**
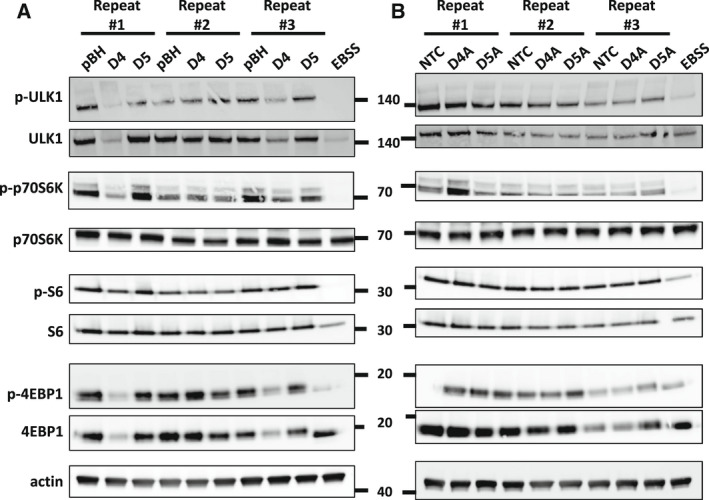
Over‐expression or deletion of DRAM‐4 and DRAM‐5 does not affect mTOR signaling. (A–B) Immunoblotting analysis of phospho‐ULK1 (Ser757), ULK1, phospho‐p70S6K, p70S6K, phospho‐S6, S6, phospho‐4EBP1, 4EBP1 in Saos2 overexpressing DRAM‐4 or DRAM‐5(A) and MDA‐MB‐157 cells in which DRAM‐4 or DRAM‐5 had been disrupted by CRISPR/Cas‐9 (B). Actin expression was used as the loading control. Starved cells (EBSS) were used as a control for mTORC1 activity.

We report here two new members of the DRAM family that exhibit both similar and also distinct functions when compared to the three previously described family members DRAM1‐3. Different to DRAM‐1, but similar to DRAM‐2 and DRAM‐3; DRAM‐4 and DRAM‐5 are not induced by the tumor suppressor p53. Instead DRAM‐4 and to a lesser extent, DRAM‐5 are induced by culture in EBSS, which lacks amino acids. Interestingly, the nutrient‐sensing kinase mTORC1 is regulated by amino acid levels and we have previously shown that DRAM‐1 has a role in this process [10]. However, our preliminary analysis indicated that neither over‐expression nor deletion of DRAM‐4 or DRAM‐5 had a reproducible effect on the phosphorylation status of mTORC1 substrates (Fig. 9).

One key characteristic of DRAM family proteins is their ability to regulate autophagy and DRAM‐4 and DRAM‐5 also impact on this process. The cells over‐expressing either DRAM‐4 or DRAM‐5 showed higher levels of LC3‐II when grown under nutrient replete conditions, but in both cases this difference was lost when the cells were cultured in EBSS for a prolonged period of time. It was interesting to discover that an up‐regulation of LC3‐II was also observed upon CRISPR‐mediated disruption of DRAM‐4. We subsequently found, however, that loss of *DRAM‐4* leads to an up‐regulation of *DRAM‐5* and that deletion of *DRAM‐5* ablates the ability of *DRAM‐4* deletion to increase LC3‐II levels. On the one hand, due to the sequence similarity between *DRAM‐4* and *DRAM‐5* it seems not surprising that there is compensatory regulation and functions between the two proteins. What is surprising, however, is that the two proteins reside in clearly distinct parts of the cell. Future studies to answer these questions are not straightforward, but would certainly be rewarding.

Another key finding from our study is that deletion of *DRAM‐4*, via *DRAM‐5*, can promote cell survival in EBSS, and the absence of serum or glucose. This effect was also observed by over‐expression of DRAM‐3 in our previous work [16]. Interestingly, DRAM‐5 localizes to the plasma membrane and DRAM‐3, localizes, at least in part, with actin‐rich focal adhesions. Does this indicate therefore that due to their over‐lapping sub‐cellular localizations that they perform similar or the even same effects to promote cell survival in the absence of glucose? Once again, this questions the potential compensatory roles between these proteins. In addition, is it possible that DRAM‐5 and DRAM‐3 also have additive roles at sites on the plasma membrane to promote either autophagy and/or cell survival?

Our expression studies showed that DRAM‐4 and particularly DRAM‐5 have varying levels of expression in cell lines from breast cancer. It is difficult based on these data to know if this is just cell line variation or if it is of significance for this disease. More extensive GWAS studies would be required to prove or discount this link. Moreover, with the function of these proteins in cell survival and with potentially extracellular or intercellular functions based on the localization of DRAM‐5, the generation of mice lacking the genes encoding DRAM‐4 and DRAM‐5 to test how loss of the genes impact breast cancer and other diseases would be worthwhile. This is clearly beyond the scope of this study, but we hope that the initial characterization of these proteins that we present here will act as inspiration for the future investigation of these interesting proteins and of the DRAM family in general.

## Materials and methods

### Cell culture

All cell lines (except for Raji, Jurkat, and HL‐60 cell lines) were grown in DMEM (Gibco, catalog no. 21969‐035, Billings, MT, USA) supplemented by 10% fetal bovine serum (FBS; Gibco, catalog no. 10270‐106), 2 mm of glutamine (Gibco, catalog no. 25030‐032), streptomycin (100 µg·mL^−1^), and penicillin (100 U·mL^−1^; Gibco, catalog no. 15140‐122) (complete DMEM) at 37 °C and 5% of CO_2_. Suspension cells (Raji, Jurkat, and HL‐60) were grown in RPMI (Gibco, catalog no. 31870) supplemented with the same reagents mentioned above at 37 °C and 5% of CO_2_. All cell lines were tested for mycoplasma (negative) and were obtained from Beatson Institute Stocks (Glasgow, UK).

For starvation experiments, the cells were washed twice in PBS and starved in complete DMEM without FBS (‐FBS), EBSS (Sigma‐Aldrich, catalog no. E2888, St. Louis, MO, USA), or glucose‐free DMEM (Gibco, catalog no. 11966) supplemented with 10% of dialyzed FBS (Gibco, catalog no. 26400044) as indicated.

Where indicated, the cells were treated with 5 µm of chloroquine (Sigma‐Aldrich, catalog no. C‐6628), 5 µm of cisplatin (Sigma‐Aldrich, catalog no. C2210000), 10 µm of etoposide (Sigma‐Aldrich, catalog no. E‐1383), 100 ng·mL^−1^ of interferon‐γ (IFN‐γ, R&D Systems, catalog no. 285‐IF‐100, USA), or 5 ng·mL^−1^ of tumor necrosis factor α (TNFα, Sigma‐Aldrich, catalog no. T0157). Tet‐on p53 Saos‐2 cells were treated with 1 µg·mL^−1^ of doxycycline (Sigma‐Aldrich, catalog no. D9891).

### Transfections and infections

The cells were transfected using calcium phosphate precipitates as previously described [20].

Retroviral infections were performed with the following constructs in Saos‐2 and Saos‐2 EcoR as previously described [10]: pBabe DRAM‐4 Hygro and pBabe DRAM‐5 Hygro. After infection, Saos‐2 cells were selected with 100 µg·mL^−1^ of Hygromycin B (Invitrogen, catalog no. 10687‐010, Waltham, MA, USA) for 7 days.

DRAM‐4‐ and DRAM‐5‐deficient MDA‐MB‐157 cells were generated using a lentiviral CRISPR construct [21]. Lentiviral infections were performed as previously described [22]. Briefly, the cells stably expressed guide RNAs and Cas9. After infection, the cells were selected with 2 µg·mL^−1^ of puromycin (Sigma‐Aldrich, catalog no. P9620) for 10 days. The following guide RNAs were used: *NTC* forward (5′‐CACCGGTAGCGAACGTGTCCGGCGT‐3′) [22], *NTC* reverse: (5′‐AAACACGCCGGACACGTTCGCTACC‐3′) [22], *DRAM‐4* forward (5′‐CACCGGCACCATATATAAGCATTGC‐3′), *DRAM‐4* reverse (5′‐AAACGCAATGCTTATATATGGTGCC‐3′), *DRAM‐5* forward (5′‐CACCGCAGTGATGGAGAACGCTGAC‐3′), and *DRAM‐5* reverse (5′‐AAACGTCAGCGTTCTCCATCACTGC‐3′). To confirm the CRISPR/Cas9‐mediated knockout, knockout‐cells were first transfected to express a Myc‐tagged DRAM‐4 and/or DRAM‐5, and the protein expression was monitored by immunoblotting. In addition, genome sequencing was performed to further validate DRAM‐4 and DRAM‐5 CRISPR knockout.

### Genomic DNA purification and sequencing

Genomic DNA of CRISPR/Cas9‐mediated knockout cells was isolated and purified using the QIAamp DNA mini Kit (Qiagen, catalog no. 51304, Germantown, MD, USA) according to manufacturer’s instructions. The DRAM‐4 and DRAM‐5 sequences targeted by the CRISPR constructs were amplified by PCR, using 20 ng of genomic DNA, and a HotStar High Fidelity Polymerase kit (Qiagen, catalog no. 202742) according to manufacturer’s instructions. PCR was performed for 40 cycles, and the PCR products were run on a 2% agarose gel. After excision, gel purification was performed using the QIAquick Gel Extraction Kit (Qiagen, catalog no. 28706X4) according to manufacturer’s instructions. The purified product was then sequenced.

The following primers were used: *DRAM‐4* forward (5′‐ATATGAATTAGTGCAGTTAG‐3′), *DRAM‐4* reverse (5′‐ACATCGTATGATACTAAATG‐3′), *DRAM‐5* forward (5′‐GAGCGCTGGATAAGGTGTTG‐3′), and *DRAM‐5* reverse (5′‐CCTGCTGGACAGCAGTGGTG‐3′).

### Reverse transcription quantitative polymerase chain reaction (RT‐qPCR)

RNAs were extracted from the cells using the RNeasy Mini Kit (Qiagen, catalog no. 74101) according to the manufacturer’s instruction and quantified using a NanoDrop200c (Thermo Fisher Scientific, Waltham, MA, USA). Complementary DNAs (cDNAs) were produced using the High‐Capacity RNA‐to‐cDNA Kit (Thermo Fisher Scientific, catalog no. 4388950) according to the manufacturer’s instruction. Quantitative polymerase chain reactions (qPCRs) were performed using the DyNAmo SYBR Green qPCR Kit (Thermo Fisher Scientific, catalog no. F‐410) on a Step‐One Plus (Applied Biosystems, Waltham, MA, USA) as follows: 20 s at 95 °C, followed by 40 cycles of 3 s at 95 °C, and 30 s at 60 °C. Methods adapted from our previous work [23]. mRNA quantification was calculated using the 2^−ΔΔCT^ method. The following primers were used: *CDKN1A* (Qiagen, catalog no. QT00062090), *DRAM‐1* forward (5′‐GCCACATACGGATGGTCATCTCTG‐3′), *DRAM‐1* reverse (5′‐GTGACACTCTGGAAATCTTGGGAT‐3′), *DRAM‐4* forward (5′‐GATGGGAAGAAATGCAGCG‐3′), *DRAM‐4* reverse (5′‐CCAGGTTTCCTTTCAGCTG‐3′), *DRAM‐5* forward (5′‐GGATCATGCCAGGTCTCTG‐3′), *DRAM‐5* reverse (5′‐GCGATGACAGCCAGCACAC‐3′), *18S* forward (5′‐GTAACCCGTTGAACCCCATT‐3′), and *18S* reverse (5′‐CCATCCAATCGGTAGTAGCG‐3′).

### Protein extraction and immunoblotting

Protein extraction was performed as previously described [10]. Protein lysates were separated by SDS/PAGE and blotted onto PVDF membranes as previously described [10]. For DRAM‐4 and DRAM‐5 detection, protein samples were deglycosylated using the PNGase F deglycosylation kit (New England Biolabs, catalog no. P0704, Ipswich, MA, USA) according to the manufacturer’s instructions. The following antibodies were used at a dilution of 1 : 1000 unless otherwise stated: p53 DO1 (BD Bioscience, catalog no. 554293, Franklin Lakes, NJ, USA, RRID: AB_395348), LC3B (Cell Signaling Technology, catalog no. 2775, Danvers, MA, USA, RRID: AB_915950), Myc‐tag (Millipore, catalog no. 05‐724, Darmstadt, Germany, RRID: AB_309938), glyceraldehyde‐3‐phosphate dehydrogenase (GAPDH; Abcam, catalog no. ab9485, Cambridge, UK, RRID: AB_307275), β‐actin (Cell Signaling Technology, catalog no. 4970, RRID: AB_2223172), extracellular signal‐regulated kinase 2 (ERK2; Santa Cruz Biotechnology, catalog no. sc‐154, Dallas, TX, USA, RRID: AB_2141292), phospho‐p70S6K (Cell Signaling Technology, catalog no. 9234, RRID: AB_2269803), p70S6K (Cell Signaling Technology, catalog no. 2708, RRID: AB_390722), phospho‐ULK1 Ser 757 (Cell Signaling Technology, catalog no. 6888, RRID: AB_10829226), ULK1 (Cell Signaling Technology, catalog no. 8054, RRID: AB_11178668), phospho‐S6 (Cell Signaling Technology, catalog no. 4858, RRID: AB_916156), S6 ribosomal protein (Cell Signaling Technology, catalog no. 2317, RRID: AB_2238583), phospho‐4EBP1 (Cell Signaling Technology, catalog no. 2855, RRID: AB_560835), 4EBP1 (Cell Signaling Technology, catalog no. 9644, RRID: AB_2097841) anti‐rabbit IgG HRP‐linked (Cell Signaling Technology, catalog no. 7074, RRID: AB_2099233; 1 : 4000), and anti‐mouse IgG HRP‐linked (Cell Signaling Technology, catalog no. 7076, U. S. National Institutes of Health, Bethesda, MD, USA, RRID: AB_330924; 1 : 4000). Protein level in blots was quantified by densitometry using imagej.

### Immunofluorescence (IF)

The cells were plated on glass coverslips. After 2 days, coverslips were washed once in PBS and then fixed with 4% para‐formaldehyde for 30 min at room temperature. Immunofluorescence staining of cells was carried out as previously described [24]. The following antibodies were used for IF analyses: Myc‐Tag (Millipore, catalog no. 05‐724, RRID: AB_309938), Myc‐Tag (Cell Signaling Technology, cat no. 2272, RRID: AB_10692100), LC3B (Cell Signaling Technology, cat no. 2775, RRID: AB_915950), EEA1 (Abcam, cat no. ab2900, RRID: AB_2262056), Calnexin (Cell Signaling Technology, cat no. 2679, RRID: AB_2228381), E‐cadherin (Cell Signaling Technology, cat no. 3195, RRID: AB_2291471), γ‐catenin (Cell Signaling Technology, cat no. 2309, RRID: AB_823448), COX IV (Abcam, cat no. ab16056, RRID: AB_443304), Alexa Fluor 488® goat anti‐mouse IgG (Molecular Probes, cat no. A‐11001, USA, RRID: AB_2534069), Alexa Fluor 488® goat anti‐rabbit IgG (Molecular Probes, cat no. A‐11008, RRID: AB_143165), Texas‐Red® goat anti‐mouse IgG (Thermo Fisher Scientific, cat no. T‐6390, RRID: AB_2556778), and Texas‐Red® goat anti‐rabbit IgG (Thermo Fisher Scientific, cat no. T‐6391, RRID: AB_2556779). Images were obtained on a Zeiss 710 confocal microscope at a × 63 magnification and quantified using the coloc2 package in Fiji (imagej).

### Clonogenic assays

Five thousand cells were seeded in 6‐cm dishes and left to attach overnight before starvation. The next day, the cells were washed two times with PBS and then incubated with starvation medium for 24 h. Medium was then exchanged back to complete DMEM and cells were left growing for further 7–10 days.

The cells were washed with PBS once and then incubated with Giemsa stain (Sigma‐Aldrich, GS500) for 15 min. The stained cells were washed four times with 10% of methanol and left to dry overnight.

### Sequence alignment

Alignment of multiple peptide sequences was performed using clustal omega (EMBL‐EBI, European Bioinformatics Institute, University College Dublin, Dublin, Ireland).

### Statistics

Statistical analysis of data was performed using the graphpad prism software (GraphPad, San Diego, CA, USA). Statistical tests used to analyze the data are indicated in the corresponding figure legends. Results were considered statistically significant when *P* value < 0.05 (*), *P* value < 0.01 (**), *P* value < 0.001, or *P* value < 0.0001 (****), with ns indicating no significance.

## Conflict of interest

The authors declare no competing interests.

## Author contributions

VJAB, MM, and KMR conceived the study and designed experiments. VJAB, MM, EK, DGMcE, DC, JOP, and JSL conducted and analyzed experiments. VJAB and KMR wrote the manuscript.

### Peer review

The peer review history for this article is available at https://publons.com/publon/10.1111/febs.16365.

## Data Availability

The data that support the findings of this study are available on request from the corresponding author.
